# Role of Pregabalin to Decrease Postoperative Pain in Microdiscectomy: A Randomized Clinical Trial

**DOI:** 10.7759/cureus.12870

**Published:** 2021-01-22

**Authors:** Shahzaib R Baloch, Imtiaz A Hashmi, Mohammad S Rafi, Ambreen Wasim, Saddam Mazar, Nadia Malick, Banin Tayyab, Hoordana Riaz

**Affiliations:** 1 Orthopaedics, Dr. Ziauddin Hospital, Karachi, PAK; 2 Orthopedics/ Consultant Spine and Orthopedics Surgeon, Agha Khan University Hospital, Karachi, Karachi, PAK; 3 Orthopedics Department/ Spine and Orthopedic Surgeon, Dr. Ziauddin Hospital, Karachi, PAK; 4 Department of Research , Ziauddin University, Karachi, PAK; 5 Orthopaedic Surgery, Dr. Ziauddin Hospital, Karachi, PAK; 6 Department of Internal Medicine, Dr. Ziauddin Hospital, Karachi, PAK; 7 Orthopedics/Intern, Dr. Ziauddin Hospital, Karachi, PAK; 8 General Surgery, Bolan Medical Complex Hospital, Quetta, PAK

**Keywords:** prolapsed intervertebral disc, microdiscectomy, pregabalin, neuropathic pain, postoperative pain

## Abstract

Purpose

The purpose of this study is to compare the effect of pregabalin in reducing the neuropathic pain in postoperative patients who have undergone single-level microdiscectomy for prolapsed intervertebral lumbar disc.

Methods

A randomized control clinical trial was conducted from June 2018 to April 2020 in three campuses Dr. Ziauddin University Hospital, Karachi, by two spinal surgeons. This study included 84 patients who underwent either emergency or elective microdiscectomy surgery. The patients randomized into two equal groups of 42, (group-A: pregabalin) and (group-B: placebo). Both groups also received routine analgesia along with the pregabalin and placebo capsules. In the intervention group, pregabalin was administered preoperative and postoperative defined times. The pain scores were recorded by visual analog scale (VAS) and Roland-Morris score system on the preoperative day and compared to the scores on follow-up on postoperative day seven.

Results

The pain scores were significantly better in group-A compared to group-B with similar baseline variables. The mean VAS scores of pains in group-A on postoperative day seven on follow-up were compared to VAS pain scores in group-B showing better pain control. The Roland-Morris scores were also significantly better for group-A.

Conclusions

The use of pregabalin in addition to the routine analgesia has better control of postoperative neuropathic pain in patients with single-level microdiscectomy compared to the patients who are receiving only routine analgesia. Other factors like cost, dose, side effects, and frequency should also be considered.

## Introduction

Lower back pain with severe radiculopathy is caused by the herniation of the lumbar disc into the spinal column with nerve root compression. This herniation can lead to devastating outcomes if not treated. In case of complications of untreated prolapsed discs like cauda equina syndrome, urgent surgical intervention is required. A minimally invasive approach to this surgery like "microdiscectomy" is necessary to relieve pain and to prevent further progression of damage.

Discectomy is a routine procedure performed by orthopedic surgeons and neurosurgeons, and it is associated with mild to moderate levels of postoperative pain. Multimodal treatments have been used to reduce side effects and doses to achieve adequate analgesic effects [1]. Treatment of postoperative pain poses significant challenges in postoperative care due to ethical, medical, and humanitarian reasons as proposed by Kehlat et al. [1] and it is thus crucial to manage it efficiently, using a multimodal approach for optimum recovery response, as endorsed by the American Society of Anesthesiologists (ASA) [2].

As the number of day-care procedures has increased by a significant number globally, newer, and more complex methods are being devised almost daily. While this may have its own benefits [3], these procedures may cause the patient to experience more pain in the initial postoperative period due to surgical trauma induced hyperalgesia [4]. Despite that, microdiscectomies are being performed in ambulatory settings on a regular basis. While initially, the standard method for preventing and treating acute postoperative pain was by administering opioid analgesics, it has, in recent times, been replaced by a combination of nonopioid analgesic drugs with different mechanisms of action as part of a multimodal approach to prevent pain [5,6].

Almost 80% of patients still suffer from postoperative pain despite all the recent advances [7] which pose as a threat towards the road of recovery of the patient, both physically as well as mentally, and thus prolonging the time to recover and achieve milestones, whilst occasionally lengthening the hospital stay, thereby making the patient more vulnerable to acquiring opportunistic infections, amongst other postoperative complications [8].

It is often noted that the risk of inappropriate pain control in patients undergoing surgery, has been relatively higher in patients who are suffering from chronic back pain, as they have already developed a tolerance to strong analgesic [9]. One of the possible reasons for the poor response to analgesics could be due to the loss of opioid receptors at the site of compression/peripheral nerve pathology. However, increasing the dosage of the opioid has proven to show adequate pain control, although this may lead to tolerance and subsequent dependence on the drug, and a phenomenon known as opioid-induced hyperalgesia [9].

It has been noted that administration of epidural analgesia has shown promising results in significantly reducing postoperative pain after spinal surgeries [10]. It has also been proven, by Mathiesen et al, that a multimodal approach prevents the usage of opioids for postoperative pain management, thereby reducing the potential side effects and addictive properties of said agents [11], especially when the dosage of pregabalin is kept >300mg as suggested by a meta-analysis carried out by Lam et al. [12].

When discussing the management of acute and/or chronic states of pain, such as diabetic peripheral neuropathy, gabapentin has not shown to have much significance over other agents, such as tricyclic antidepressants (amitriptyline), and thereby are often used alone or in combination depending on the condition of the patient and their comorbidities [13], however, pregabalin has shown to have a much superior pharmacokinetic profile than its precursor [14].

Pregabalin is structurally a gamma-aminobutyric acid (GABA) and has the same characteristics and mechanism of action as its precursor gabapentin [14]. For managing and treating acute pain, the efficacy of pregabalin is similar to that of gabapentin; however, having a greater bioavailability, linear pharmacokinetics, lower dose titration, and a shorter-acting time to achieve optimal analgesic effect makes pregabalin more superior to its precursor [14,15]. Pregabalin also appears to have a higher success rate for managing acute nociceptive pain postoperatively, and thereby reducing opioid-induced dependence and anxiety. [14,16,17]

Pregabalin has been administered as part of analgesic regimens for various procedures, including lumbar spine surgery, in a few studies. One such study conducted by Mohsin et al., revealed, the effectiveness of both agents (gabapentin and pregabalin), for postoperative management for single-level microdiscectomy was almost equivalent. [18]

In other studies, pregabalin was either compared to a placebo or there were limitations in the study design, with respect to the pharmacodynamics of the drug, such as it being utilized just before the surgery, instead of administering it at least a week prior in order to produced successful results, as seen in reality [14,19]. Thus, in order to achieve maximal benefits, interventional drugs should be administered one week prior to the actual procedure, as the preemptive use of GABA analogs preoperatively reduces not just the side effects, but the requirement for postoperative analgesia as well.

Therefore, we intended to compare the postoperative pain control in patients with the usage of low dose pregabalin along with routine analgesia to those with patients who received only routine analgesia pre and postoperatively.

We hypothesized that the pain in the pregabalin group would be less than that in the without pregabalin group.

## Materials and methods

This study is a single-blinded, parallel-group, randomized clinical trial that was conducted in the orthopedics department of Dr. Ziauddin University Hospital Karachi. Conducted on patients who were suffering from chronic sacral radiculopathy and diagnoses to have PID (prolapsed intervertebral disc), undergoing elective lumbar microdiscectomy. The study started with IRB (institutional review board) approval and written/verbal informed consent from the patients. Then they were randomly allotted in two groups: group-A (pregabalin group) and group-B (control group). Each group consisting of 42 patients each with a total of 84 patients, aged between 27 years to 61 years. The exclusion criteria included patients with previous lumbar surgery, patients allergic to gabapentinoids, patients with renal impairments, patients already on opioids, benzodiazepines, barbiturates, ethanol (alcohol), diabetes, and other drugs that depress the central nervous system and on angiotensin-converting enzyme (ACE) inhibitors as they may enhance the adverse/toxic effect of pregabalin. 

In this study, the patients received pregabalin 75 mg or placebo (sucrose) twice daily from preoperative day till one week of surgery. All medications were identical and provided by the hospital pharmacy in sealed opaque envelopes. Along with this, all patients were getting regular postoperative pain management with paracetamol 1 gram six hourly and nonsteroidal anti-inflammatory medication (Ketorolac) 30 mg I/V ter die sumendum (TDS) as a primary protocol for pain management. Opioids were given on pro re nata (PRN) bases if the pain was not controlled via the primary protocol. All patients were given three pain scale questionnaires Roland-Morris disability scale and visual analog scale (VAS) pain to fill, preoperatively, and then after one week of surgery, and the results were compared.

Data was analyzed using Excel 2013 and IBM SPSS Statistics ver.20 (SPSS Inc., Chicago, IL, USA). Contingency tables were analyzed using Fisher exact test, P values <0.05 were considered statistically significant.

## Results

**Figure 1:**
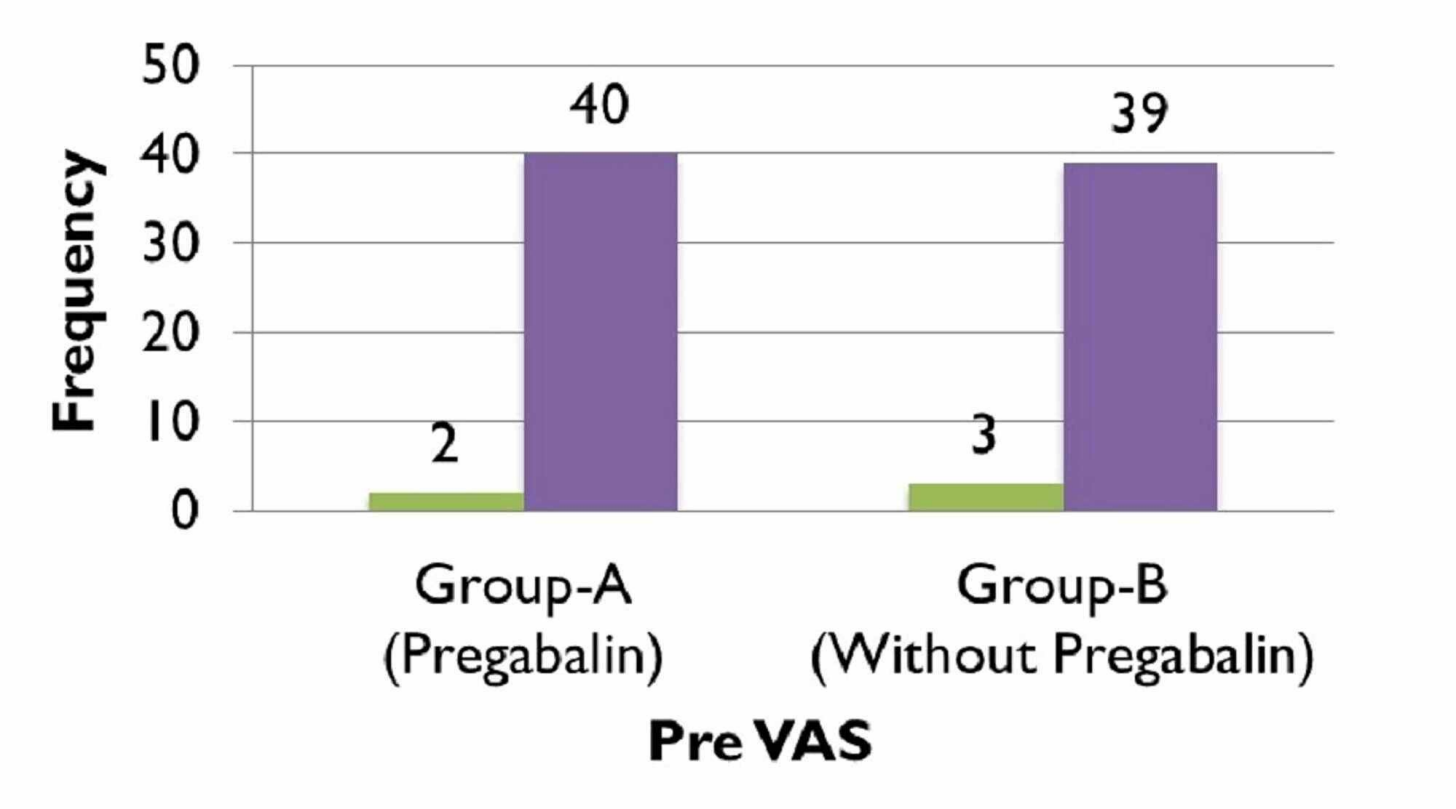
Preoperative visual analog scale (VAS) scores with comparison groups Pearson Chi-square = 0.213, p=0.645; *Significant at p<0.05

**Figure 2:**
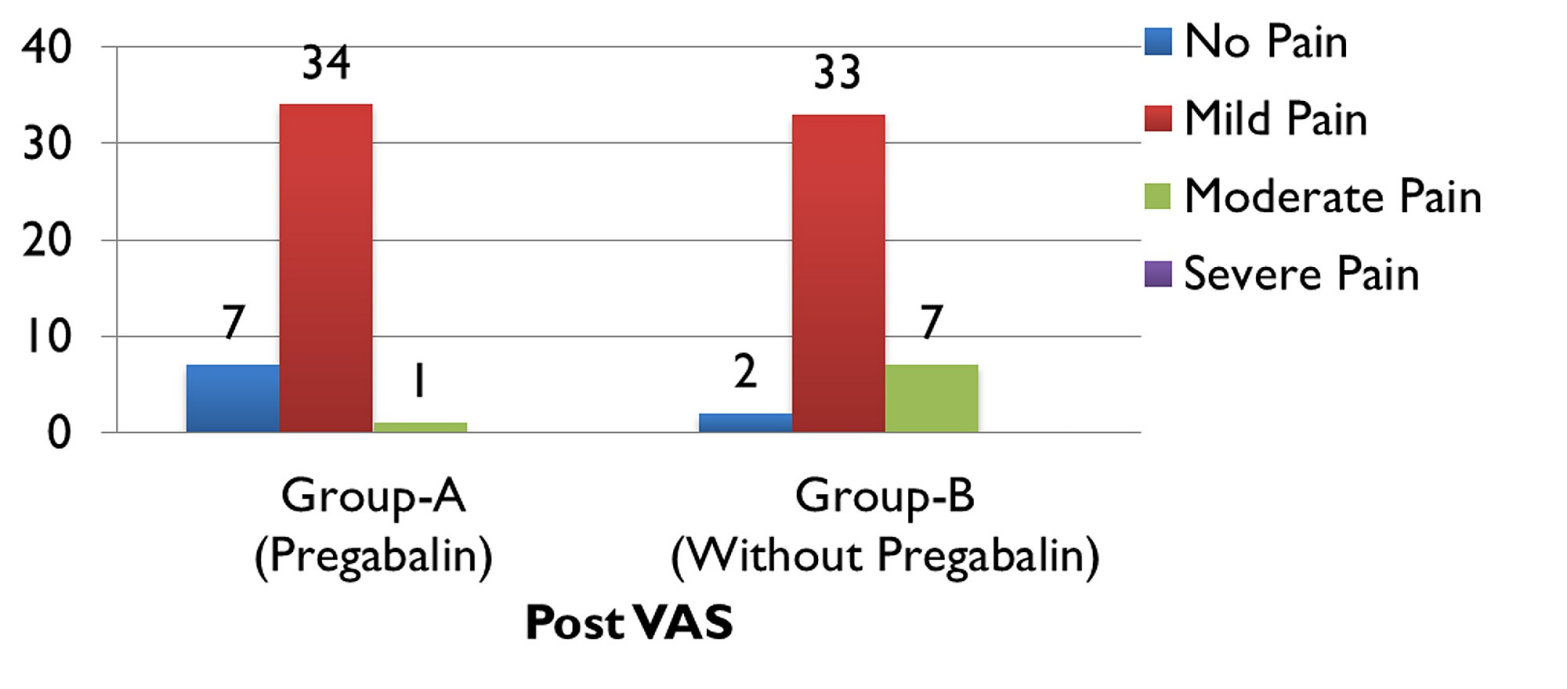
Postoperative visual analog scale (VAS) score with comparison groups Pearson Chi-square = 7.293, p=0.026*; * Significant at p<0.05

**Figure 3:**
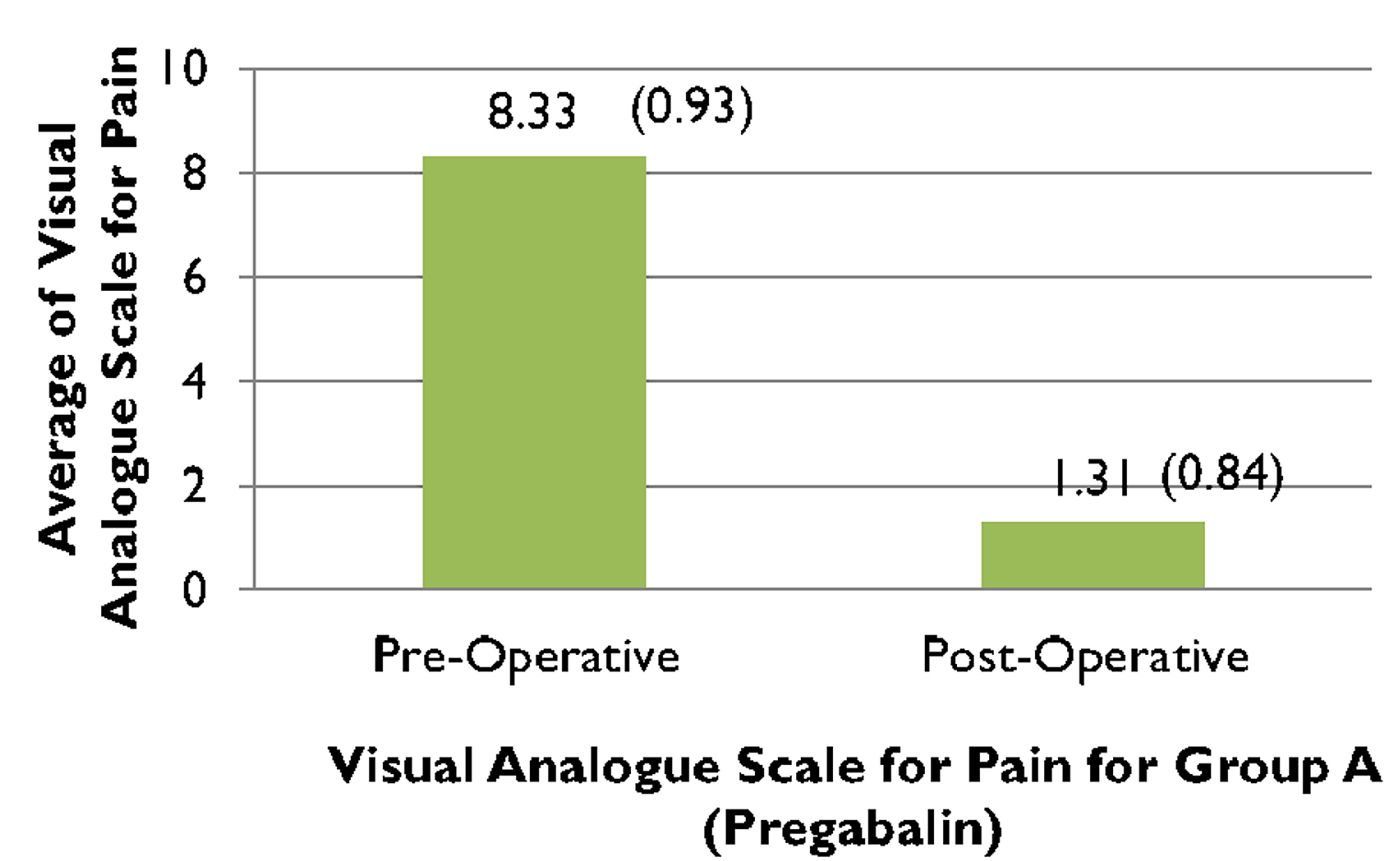
Comparison between preoperative and postoperative visual analog scale (VAS) scores for pain for group-A Paired t-test = 52.369, df =41 p<0.0001*

**Figure 4:**
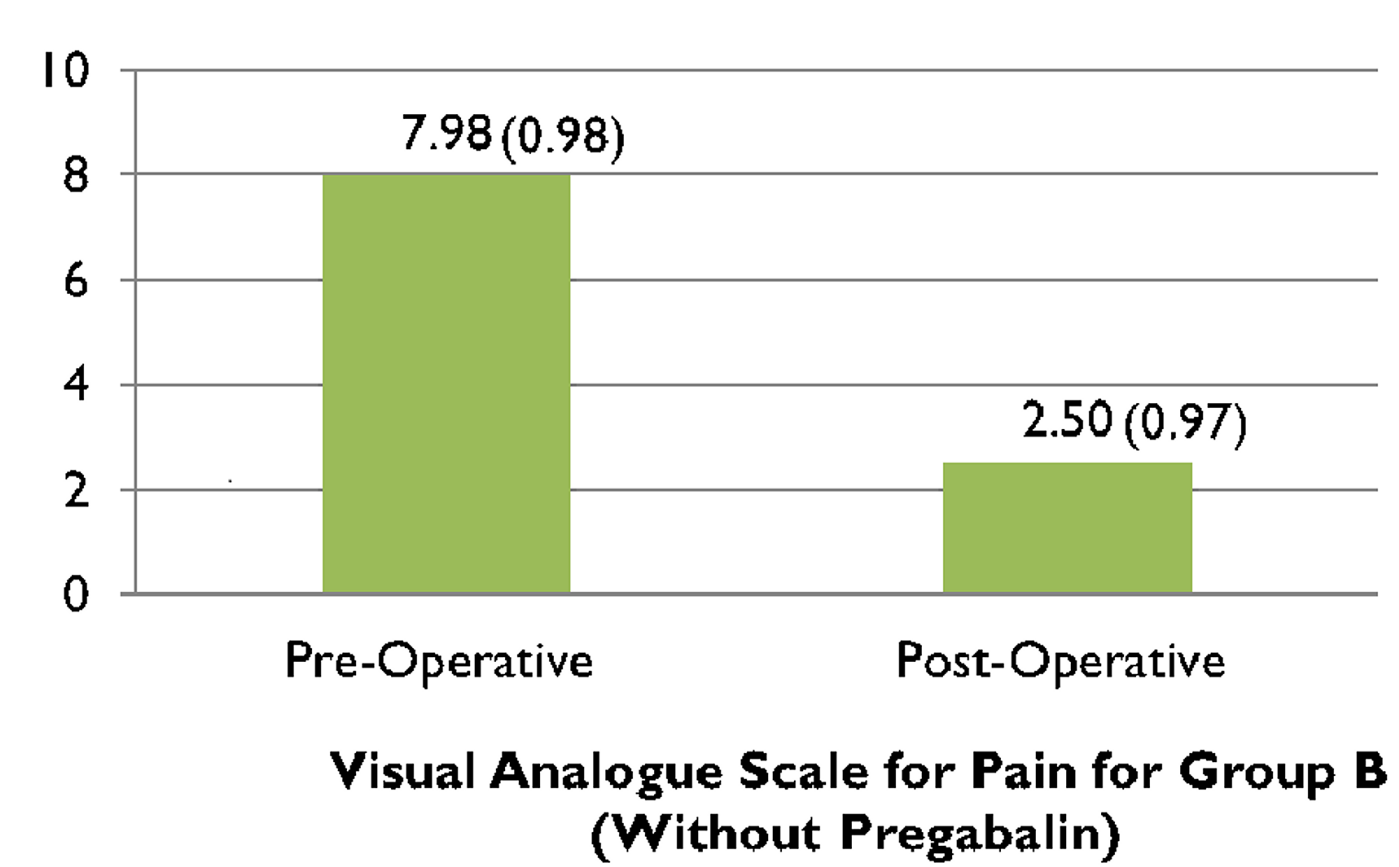
Comparison between preoperative and postoperative visual analog scale (VAS) scores for pain for group-B Paired t-test = 24.725, df =41 p<0.0001*; * Significant at p<0.05

**Figure 5:**
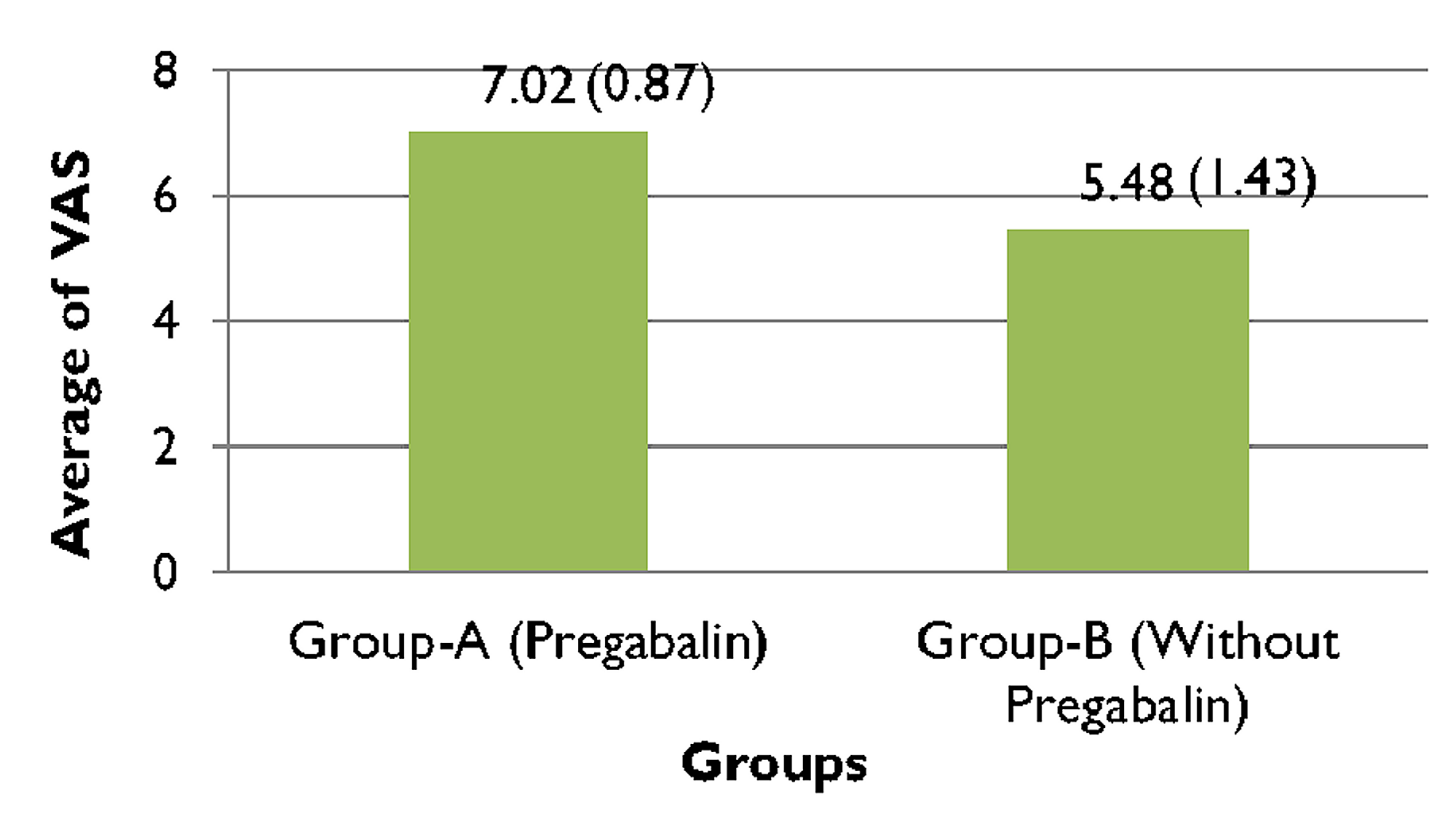
Average visual analog scale (VAS) scores comparisons between group-A and group-B Independent Sample t-test = 5.977, df =67 p<0.0001*; * Significant at p<0.05

Our study comprised of 84 patients, equally divided into two groups based on random selection; group A consisted of 42 patients, who received pregabalin pre and postoperatively, and group B consisted of the remainder of patients who did not receive pregabalin. Amongst our participants, 58% (n=49) of participants were females, while the remaining 42% (n=35) were males. The age of the participants ranged between 27 years to 61 years, categorizing them as adults, and thus all the participants received the standardized adult dosage of the analgesics (including pregabalin for group A only). For the assessment of pain, two international scoring systems; the 10-point VAS and the 24-item patient-reported Roland-Morris scoring system for lower back pain (RMS), were used. The pain was assessed both preoperatively and one week postoperatively, in both groups. Keeping the significance of P at <0.05, the results of our research are as follows:

When comparing the pre and post-operative VAS scores of group A, (Figure 1), 95.2% (n=40) patients complained of experiencing severe pain preoperatively which significantly improved post-operatively after receiving treatment with pregabalin (along with other analgesic agents) and reduced to 2% of participants of group A (n=1) experiencing moderate pain, while almost 81% of patients (n=34) endured mild pain and nearly 16% (n=7) felt no pain (Figure 2). In contrast to that, just about 93% of patients (n=39) in group B experienced severe pain preoperatively (Figure 1), which reduced to only 16% (n=7) feeling moderate pain, nearly 79% (n=33) enduring mild pain and around 4% (n=2) of patients experiencing no pain (Figure 2). The preoperative VAS score for group A was 8.33, which significantly lowered to 1.31 postoperatively, seen in (Figure 3). Whereas (Figure 4) shows that the group not receiving pregabalin (group B), the preoperative VAS amounted to 7.98 which reduced to 2.50 postoperatively. The average VAS for group A totaled 7.02, while for group B it came to 5.48, (Figure 5). In either of the groups, no patients reported severe pain, postoperatively, regardless of the administration of pregabalin.

**Figure 6:**
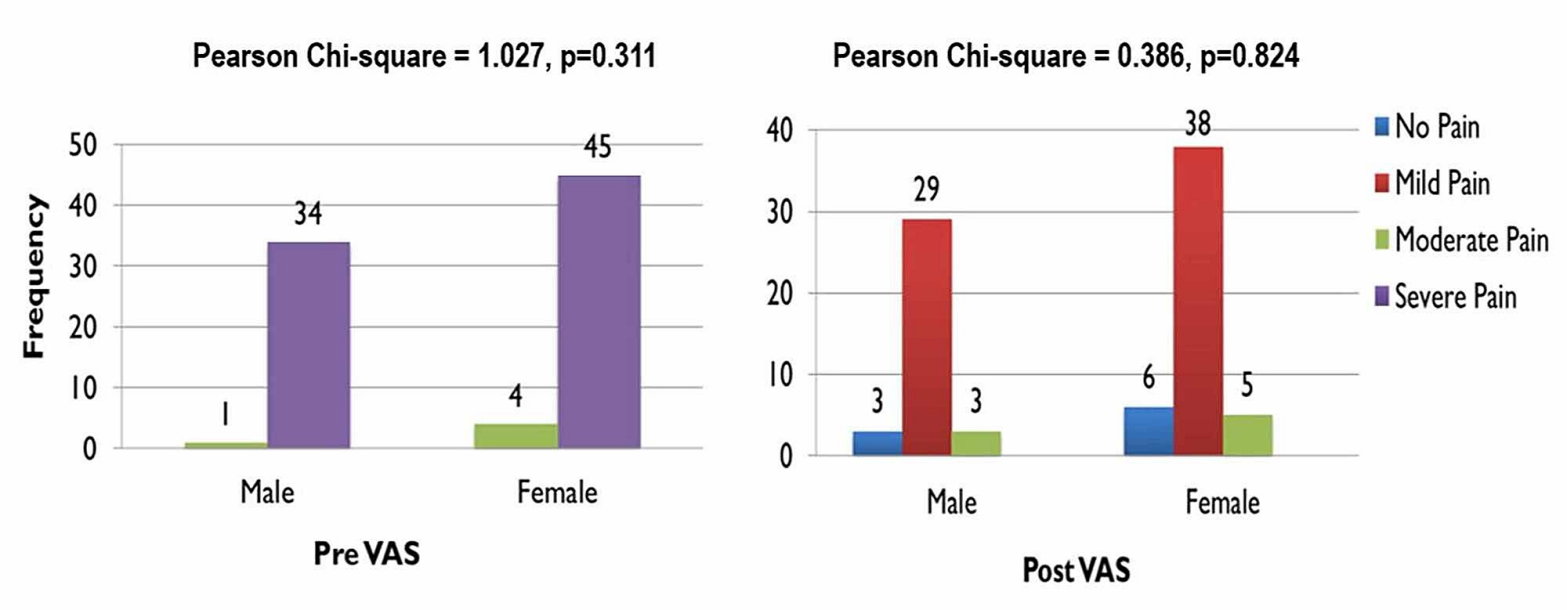
Gender wise comparison between pre and postoperative visual analog scale (VAS) scores Significant at p<0.05

**Figure 7:**
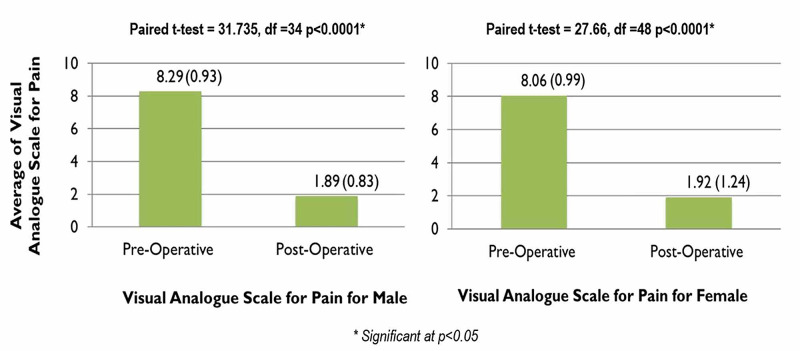
Comparison between pre & postoperative visual analog scale (VAS) scores in both males and females

**Figure 8:**
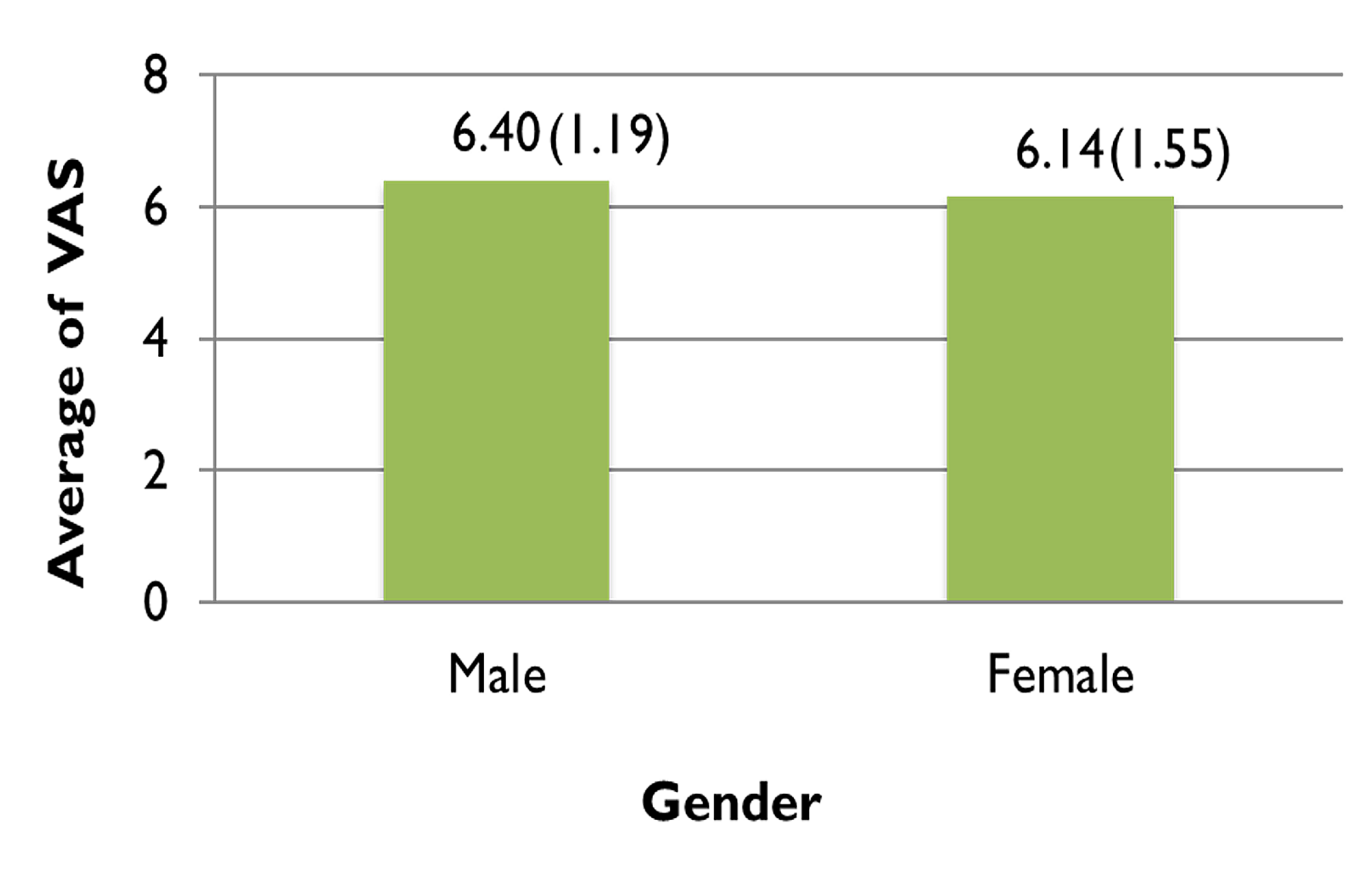
Average of visual analog scale (VAS) scores in males and females Independent Sample t-test = 0.812, df =82 p=0.414

Pain relief was almost comparable in both genders, with almost 83% (n=29) of males enduring mild pain and only 8% (n=3) enduring moderate pain postoperatively, while 77% (n=38) of females experienced mild pain and almost 10% (n=5) of them feeling moderate pain. Neither gender reported severe pain postoperatively (Figure 6). For males, the VAS equaled 8.29 preoperatively which considerably dropped to 1.89 postoperatively. Females reported an almost similar score, with their preoperative VAS equaling 8.06 and their postoperative score being 1.92, shown in Figure 7. Figure 8 demonstrates the average VAS for both genders was almost comparable, with the scores being 6.40 and 6.14 for males and females respectively.

**Figure 9:**
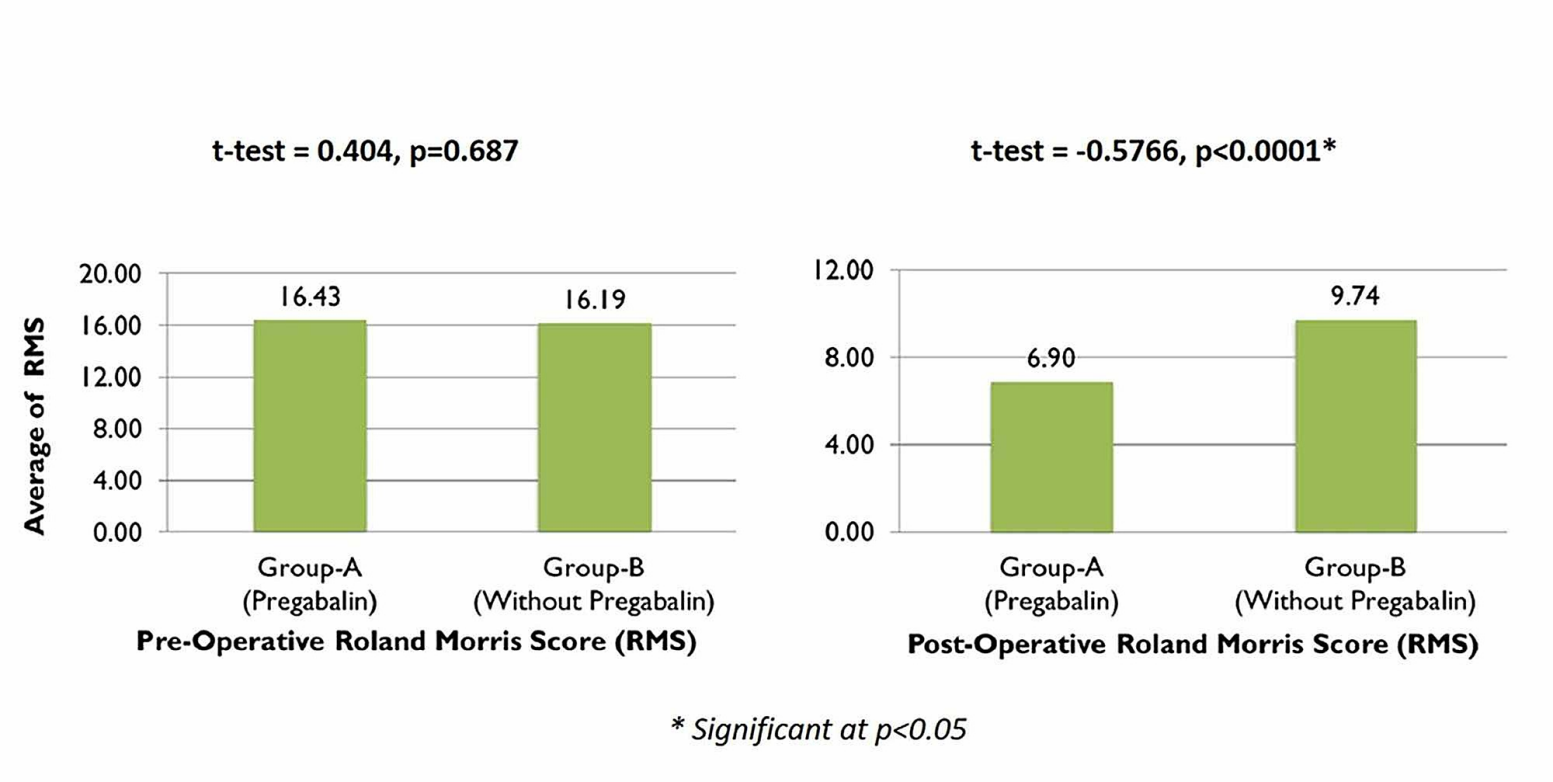
Comparisons of preoperative and postoperative Roland-Morris scores

The average RMS for group A, who were administered pregabalin pre and postoperatively, was 16.43 preoperatively, which considerably reduced to 6.90 postoperatively. However, for group B the scores did not show a great improvement, with the score being 16.19 preoperatively going down to only 9.74 postoperatively (Figure 9).

**Figure 10:**
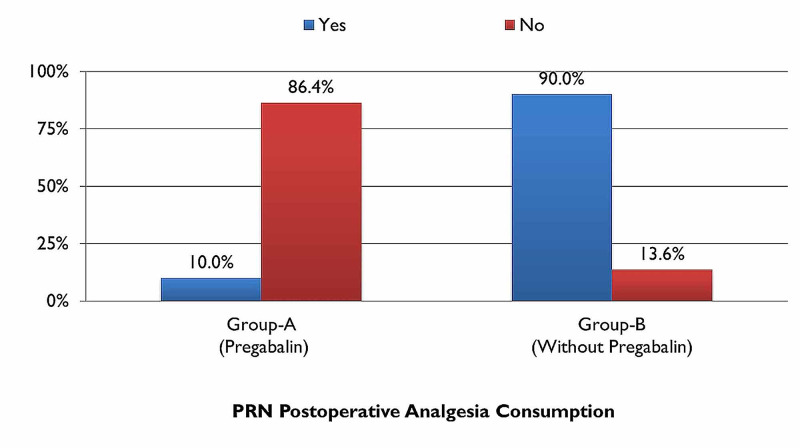
PRN (Pro Re Nata) postoperative analgesia consumption with comparison groups

There was also a notable difference between the two groups for PRN consumption of analgesics postoperatively, which, for group A was only 10% (n=4) while that of group B was 90% (n=38) as illustrated in (Figure 10), thereby aiding in confirming the hypothesis.

## Discussion

The study revealed that the pre and postoperative administration of the combination of pregabalin and regular analgesia resulted in improved pain control compared with regular analgesic drug alone after single-level microdiscectomy for prolapsed intervertebral lumbar disc.

The combination of the two drugs produced a significant reduction in pain scores (VAS score: 7.02; statistical significance of: 0.87, p value: <0.0001*) compared to regular analgesia (VAS score 5.48; statistical significance of: 1.43, p-value: <0.0001*) (significance of p= <0.05).

It has been noted that pregabalin has been more effective in providing analgesic effects for neuropathic pain, [14,20] such as in postherpetic neuralgia [21], lower limb orthopedic surgery [22], and post-Lichtenstein herniorrhaphic pain [23].

Pregabalin exhibits its anticonvulsant, analgesic, and anxiolytic activity by binding to the ligand for the alpha-2-delta subunit of voltage-gated calcium channels in the central nervous system which thereby reduces depolarization-induced calcium influx along with a modification in the release of excitatory neurotransmitters. Pregabalin inhibits the release of neurotransmitters including glutamate, noradrenaline, calcitonin gene-related peptide, and substance P [24]. The reduction in a combination of several neurotransmitters is responsible for decreased pain scores as observed in our study.

The extensive and rapid absorption of pregabalin is proportional to the dose. Maximal plasma concentration is attained in approximately one hour while steady-state is accomplished within 24-48 hours which shows that the observed onset of efficacy for pregabalin, is as early as day two in most clinical trials. The mean elimination half-life is calculated to be around 6.3 hours. These aforementioned factors predict a dose-response relationship in clinical practice, thereby allowing an effective starting dose of 150mg/day to be administered without any need for titration [14].

In our study, we administered pregabalin to our patients preoperatively, approximately (24) hours before the surgery. As a result, it is expected that the peak plasma concentration for pregabalin was achieved.

We also found in our study that preoperative pregabalin decreased usage of postoperative opioid use for pain management significantly. (P-value <0.0001). Our results also correspond with a randomized controlled study which concluded that in the first 48 hours post-procedure, the number of doses needed for rescue analgesia, and total required dose of morphine, were significantly less (p=<0.0001) [25].

A study conducted in Denmark on women undergoing hysterectomy concluded that by administering spinal or epidural anesthesia, there is blockade of the central impulse traffic, that produces a protective effect towards the development of chronic pain, eventually preventing extreme postoperative discomfort [26,27]. There is also data supporting the use of spinal anesthesia over general anesthesia, in gynecological procedures such as cesarean sections, for reducing postoperative pain [27,28].

In another study conducted in March 2017 by Liu B, et al. it was concluded that when patients undergoing spinal surgery, were administered gabapentinoids preoperatively, they had a significant reduction in postoperative pain, total consumption of morphine, and morphine-related complications postoperatively [29]. Gabapentin has also shown a rapid response in multimodal analgesia [30].

## Conclusions

The use of pregabalin in addition to the routine analgesia has better control of postoperative neuropathic pain in patients with single-level microdiscectomy compared to the patients who are receiving only routine analgesia. Other factors like cost, dose, side effects, and frequency should also be considered.
